# Supramolecular Caffeic Acid and Bortezomib Nanomedicine: Prodrug Inducing Reactive Oxygen Species and Inhibiting Cancer Cell Survival

**DOI:** 10.3390/pharmaceutics12111082

**Published:** 2020-11-11

**Authors:** Ludwig Erik Aguilar, Se Rim Jang, Chan Hee Park, Kang Min Lee

**Affiliations:** 1 Department of Bionanosystem Engineering, Jeonbuk National University, Jeollabuk-do 54896, Korea; leaguilar@jbnu.ac.kr (L.E.A.); tpfla6020@gmail.com (S.R.J.); 2 Department of Bionanotechnology and Bioconvergence Engineering, Graduate School, Jeonbuk National University, Jeollabuk-do 54896, Korea; 3 Division of Mechanical Design Engineering, Jeonbuk National University, Jeollabuk-do 54896, Korea; 4 Department of Molecular Biology, College of Natural Science, Jeonbuk National University, Jeollabuk-do 54896, Korea

**Keywords:** caffeic acid, bortezomib, phenolics, cancer treatment, prodrug nanomedicine

## Abstract

Phenolics from plant materials have garnered attention in nanomedicine research, due to their various medicinal properties. Caffeic acid, a phenolic compound that is abundant in coffee beans, has been proven to have anticancer effects, due to its reactive oxygen species (ROS)-inducing properties. Here, a supramolecular nanomedicine was designed using caffeic acid molecule and the synthetic anticancer drug bortezomib, via catechol–boronic acid conjugation and Fe(III) ion crosslinking. Bortezomib is a proteasome-inhibiting drug and its boronic acid functional group can bind to caffeic acid’s catechol moiety. By having a nanoparticle formulation that can deliver bortezomib via intracellular endocytosis, the catechol–boronic acid conjugation can be dissociated, which liberates the boronic acid functional group to bind to the 26S proteasome of the cell. The ROS-inducing property of caffeic acid also complements the bortezomib payload, as the latter suppresses the survival mechanism of the cell through NF-κB inhibition.

## 1. Introduction

Nanotechnology has been in the forefront of medicine due to its potential for treating dreadful diseases, such as cancer. The use of nanoparticles is a good strategy in cancer therapy for delivering therapeutics [1] and as a diagnostic tool due to its high specificity to target tissues and organs [2], longer circulation time, and lower toxicity and side effects [3]. There have been numerous strategies in nanoparticle synthesis, and one emerging method for creating new nanomedicine is supramolecular chemistry [4]. This cross between macromolecular chemistry and nanotechnology has recently been attracting attention in medicine, due to its potential as a method for creating stable, functional, and robust nanoparticles that are tunable for treating cancer. Supramolecular chemistry pertains to the supramolecular pharmaceutical formulation of nanoscale therapeutic agents via stimuli-reversible conjugation interactions or dynamic covalent bonding. Nanoparticle formulation for drugs is an effective approach to solving various pharmacokinetic problems that therapeutics are currently experiencing. Nanoparticles can be taken up by cells via an endo/phagocytosis route, subjecting them to acidic pH conditions. This pH gradient can be used as a stimulus to dissociate the dynamic covalent bond between macromolecules used in the synthesis of the developed nanomedicine [5].

We are intrigued by the use of phenolics for supramolecular nanoparticle synthesis, due to their functional moieties that can be conjugated to drugs and targeting ligands [6]. One suitable candidate, caffeic acid (CA), has been studied for its interesting medicinal properties, e.g., anti-inflammatory [7,8], anticancer [9], and antibacterial/antiviral effects [10,11,12]. This phenolic compound can be found in high quantities in coffee beans, and is one of the reasons coffee has antioxidant effects [13,14,15]. The chemical structure of CA comprises a carboxyl group and a catechol moiety, which can serve as a drug-conjugating site for bortezomib (BTZ). BTZ is a synthetic anticancer drug that inhibits NF-κB by binding to the 26S proteasome of cancer cells with high affinity and specificity, thereby resulting in reduced protein degradation tagging and apoptosis [16,17,18,19]. BTZ can complement the use of caffeic acid by virtue of inhibition of the survival mechanism of the cell, while CA creates intracellular DNA and protein damage via the induction of reactive oxygen species (ROS) [20,21,22].

This strategy can also answer the problem of BTZ inhibition of phenolic compounds from natural diet [23]. It has recently been discovered that dietary polyphenols can block BTZ activity by binding to the boronic acid functionality of the drug. The boronic acid functionality of BTZ plays a crucial role as the main moiety responsible for binding to the proteasome that activates NF-κB. The drug can be delivered to the cancer cell by using CA’s catechol moiety as a carrier for BTZ. Another salient feature of this strategy is that the binding of BTZ to CA is pH-reversible under acidic pH, and the cancer cell’s intracellular and tumor environment acidic condition can be exploited as a stimulus to deliver the drug payload [24], liberating BTZ from CA and ensuring that both components can enact their anticancer properties [25]. This kind of drug formulation is also known as a prodrug approach, where the drug itself, in this case the nanomedicine, cannot act as an anticancer compound, due to the inhibitory effects of the boronic acid–catechol conjugation components on each other [26]. Bortezomib and caffeic acid cannot both become anticancer agents until the nanomedicine has been metabolized inside the body through the acidic condition of the tumor and the intracellular pH of cancer cells. It is also crucial to point out that it is a new strategy by which the carrier itself (CA) can act as an anticancer agent and can help the drug payload (BTZ). Other contemporary catechol and BTZ nanomedicines rely only on BTZ as the anticancer agent without playing any other role, but CA not only acts as a carrier, but as a drug.

To make this nanomedicine stable under biological media, we used ferric ions as a crosslinker to the BTZ-CA macromolecule. The rational design of this nanomedicine can solve many problems in the clinical setting. Not only can it avoid the inhibition of BTZ by dietary polyphenols, it can enhance its effects by complementing it with ROS-induced damage by using CA as its carrier molecule.

## 2. Materials and Methods

### 2.1. Materials

Caffeic acid and dimethyl sulfoxide (DMSO) with ≥ 98.0% purity were purchased from Sigma-Aldrich (St. Louis, MO, USA), while bortezomib was procured from LC Laboratories (Woburn, MA, USA). Ferric chloride hexahydrate was obtained from Samchun Chemicals (Gangnam, Seoul, Korea). All aqueous solutions were prepared with ultrapure water purified with a Milli-Q UV-Plus water purification system (Millipore, Bedford, MA, USA). The water had a resistivity of > 1.018 MΩ cm^−1^.

### 2.2. Nanomedicine Synthesis (CAFeB)

Caffeic acid (0.25 mmol) and bortezomib (0.10 mmol) in a 2.5:1 molar ratio were dissolved in separate vials containing 100 µL of DMSO. Both solutions were then thoroughly mixed together to form the BTZ-CA conjugate. The resulting BTZ-CA solution was next added to 1 mL of ultrapure Distilled Water (DW) and mixed until a white frothy solution was created. FeCl_3_.6H_2_O (0.02 mmol) was then dissolved in 2 mL of DW, subsequently added to the BTZ-CA solution, and mixed together until a white precipitate was formed. The final solution was then transferred to a dialysis bag (3500 Da) and submerged in PBS solution (pH 7.4) for 5 h to remove unreacted components. The final yield was then lyophilized overnight and kept for future testing and characterization.

### 2.3. Nanomedicine Characterization

The CAFeB nanomedicine was characterized using bio-transmission electron microscopy (Bio-TEM; HITACHI H-7650, Tokyo, Japan), for morphological investigation, and field-emission transmission electron microscopy (FE-TEM) and scanning electron microscopy (FE-SEM) for elemental mapping. The nanoparticles were gathered in a copper grid, and then viewed at 400,000× magnification at 100 kV excitation voltage. The chemistry of the nanomedicine was analyzed by the ATR-FTIR method (Frontier, Perkin Elmer, Waltham, MA, USA). Pure CA, pure BTZ, and CAFeB were used to verify the presence of the relevant functional groups. H-NMR, B-NMR, and C-NMR were also completed to verify the conjugation of BTZ to CA using 500 mHz FT-NMR (JNM-ECZ500R, Jeol Ltd., Tokyo, Japan). To verify the drug and excipient compatibility of the nanomedicine, we conducted differential scanning calorimetry (DSC) and thermogravimetric analysis (TGA). Finally, Zetasizer (Nano-Z590, Malvern Instruments, Worcestershire, UK) analysis was performed to determine the average particle size and surface charges of the synthesized nanomedicine under different media conditions.

### 2.4. In Vitro Cell Culture Studies

Normal fibroblast (NIH-3T3) and colon carcinoma (CT26) mouse-derived cells were incubated using DMEM and RPMI 1640 (Gibco, Waltham, MA, USA), supplemented with 10% FBS and 1% penicillin. Before starting all experiments, at least three subcultures were done to optimize cell growth.

### 2.5. Cancer Cytotoxicity Evaluation

An assay using Cell Counting Kit 8 (Dojindo CCK8, Rockville, MD, USA) was conducted with CT26 (mouse colonic cancer cell line) to determine the metabolic activity of the cancer cells co-incubated with the synthesized nanoparticles. The cells were seeded using a 48-well cell culture plate at a seeding density of 16 × 10^4^ cells/well. Different concentrations of CA, BTZ, and CAFeB were prepared at 5, 15, and 30 µg/mL by creating a stock solution for each at 1 mg/mL in sterile PBS and subsequently diluting them with PBS to achieve the said concentrations. The prepared drugs were added and co-incubated with the CT26 cell line for 12 h (control group had no anticancer agent exposure). To determine the cancer antiproliferative properties of the nanomedicine, we conducted a separate CCK8 assay in a high- and low-dose-dependent manner, where CT26 cells in 90% confluency on 48-well plates were exposed to CA (21.4 mg/mL and 21.4 µg/mL), BTZ (8.56 mg/mL and 8.56 µg /mL) and compared to cells exposed to CAFeB (30 mg/mL and 30 µg/mL). This was followed by cell staining studies to observe the morphological response and ROS production of the cells on co-incubation conditions. DAPI and actin green staining were performed to visualize the nucleus, cytoskeleton morphology, and mitochondrial ROS levels of CT26 cancer cell line on samples incubated with 30 µg /mL of CAFeB. Calibur flow cytometry (FACS-Calibur, Becton-Dickenson, New Jersey, NJ, USA) using propidium iodide and annexin V (Sigma-Aldrich, St. Louis, MO, USA) staining was conducted to objectively count the necrotic and apoptotic cells after co-incubation for 12 h, using the same parameters as stated above.

### 2.6. Cell Death Mechanism Study

To verify the regulation of NF-κB and TNF-α cytokine levels in CT26 cancer cell line, western blot studies and quantitative PCR (Q-PCR) were performed. The cells were co-incubated with 30 µg/mL of CA, BTZ, and CAFeB nanomedicine for 24 h. The mRNA expression of TNF-α was confirmed using the TaqMan Universal PCR Master Mix (4304437, Thermo Fisher Scientific, Waltham, MA, USA), TaqMan primers, and probe sets that specifically target TNF-α (Mm00443258_m1) and GAPDH (Mm99999915_g1). To evaluate the protein level of NF-κB, cells were extracted by the PRO-PREP solution (Intron Biotechnology Inc., Seongnam-Si, Korea) for 20 min at 4° C. Protein concentration was confirmed through the Bradford assay. For western blotting, 30 μg of each protein sample was loaded onto nitrocellulose membranes. Primary antibodies against NF-κB (mAb 8242, Cell Signaling Technology, Danvers, MA, USA) and GAPDH (sc20357, Santa Cruz, CA, USA) were diluted to 1:1000. Mouse anti-rabbit lgG, HRP-conjugated secondary antibody (sc-2357, Santa Cruz, CA, USA) was diluted to 1:3000. The membrane was developed by enhanced luminol-based chemiluminescent substrate (WESTSAVE Gold, AbFrontier, Liestal, Switzerland) and exposed to an X-ray film. Caspase-3-mediated apoptosis was also assessed using a colorimetric assay with an ApoTarget caspase-3 kit. TUNEL (In situ Cell Death Detection Kit, Flourescein, Roche, St. Louis, MO, USA) staining was also done to visualize and count the number of DNA-cleaved cells co-incubated with CA, BTZ, and CAFeB, using the same concentration stated above.

### 2.7. In Vitro Dose-Dependent Biocompatibility Study

We conducted a CCK8 assay to assess at which dose the CAFeB prodrug would be toxic to normal fibroblast cells (NIH-3T3). Normal fibroblast cells were cultured in DMEM supplemented with 10% fetal bovine serum (FBS) and 1% penicillin. The cells were then seeded in 96-well culture plates at a seeding density of 50,000 cells/well. At confluence, different doses of CA, BTZ, and CAFeB (1, 5, 10, and 15 µg/mL) were added to the medium, and after 12 h of co-incubation, 20 µL of CCK8 (Dojindo CCK8, Rockville, MD, USA) reagent was added. After two hours of incubation at 37 °C, the optical density of the media was measured using a microplate reader at 450 nm. After DAPI staining, the morphology of the cells was viewed using fluorescence microscopy to view the cell nuclei. The results reported were the average of *n* = 3.

### 2.8. Comparison of the Intrinsic Mitochondrial ROS of NIH-3T3 and CT26 Cells

Mitosox Red (Thermofisher, Waltham, MA, USA) was used to verify the amount of reactive oxygen species in normal and malignant cells used in this study (NIH-3T3 and CT26). The cells were fixed using 4% paraformaldehyde and permeabilized using 2% Triton X. Afterwards, prepared Mitosox Red was added to the cell culture dish, and subsequently viewed using fluorescence microscopy (LSM 880 with Airyscan, Carl Zeiss, Germany). The fluorescence intensity was measured using ImageJ software.

### 2.9. Image and Statistical Analysis

All images were analyzed using ImageJ software. The results are presented as the mean ± standard deviation (SD) for *n* = 3. Statistical significance was assessed by determining the level of significance with one-way ANOVA followed by Tukey’s test for a comparison of means using OriginPro 8.5 software (* *p* < 0.05, ** *p* < 0.01) (OriginLab Corporation, Northampton, MA, USA).

## 3. Results

### 3.1. Chemistry Basis of the Synthesized Nanomedicine

**Figure 1:**
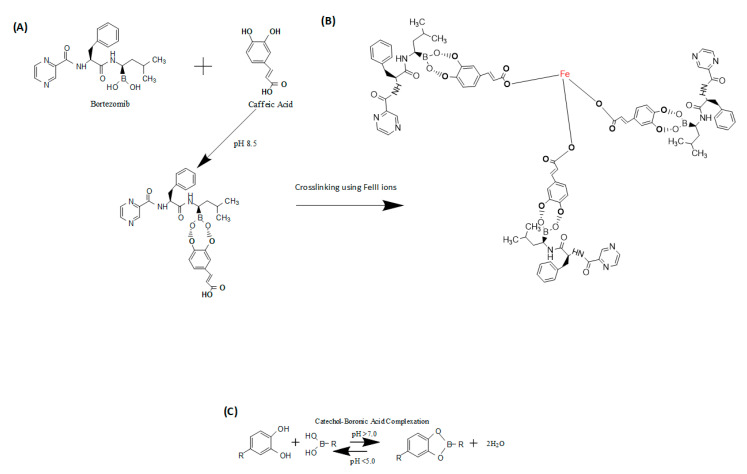
(**A**) Chemical schematics of complexation of bortezomib (BTZ) and caffeic acid (CA) to form the macromolecule BTZ-CA. (**B**) Crosslinking schematics using ferric III ions to stabilize the supramolecular prodrug nanomedicine. (**C**) Catechol–boronic acid reversible complexation and dissociation under different pH conditions.

The chemistry involved in the synthesis of the supramolecular prodrug nanomedicine involved a simple complexation of the boronic acid group of the BTZ drug and the catechol group of CA. Under alkaline conditions, this complexation happened due to the protonation of the hydroxyl (OH) groups of caffeic acid, making high affinity bonding with the boronic acid functionality of BTZ (as seen in Figure 1A). However, a stable nanoparticle with this complexation alone cannot be made, due to its polarity. A stabilizing agent is needed, and in this case, we chose to use ferric III ions from FeCl_3_ dissolved in DW. The ferric III ions covalently bound to the conjugated BTZ-CA and crosslinked the BTZ-CA macromolecules (Figure 1B). The resulting supramolecular prodrug nanomedicine will come about via the pH-reversible conjugation of the boronic acid group of the drug and catechol moiety of the phenolic compound (Figure 1C). Acidic conditions in the presence of a tumor environment can be found and used as the stimuli to liberate the drug payload.

### 3.2. Characterization of the Supramolecular Prodrug Nanomedicine CAFeB

**Figure 2:**
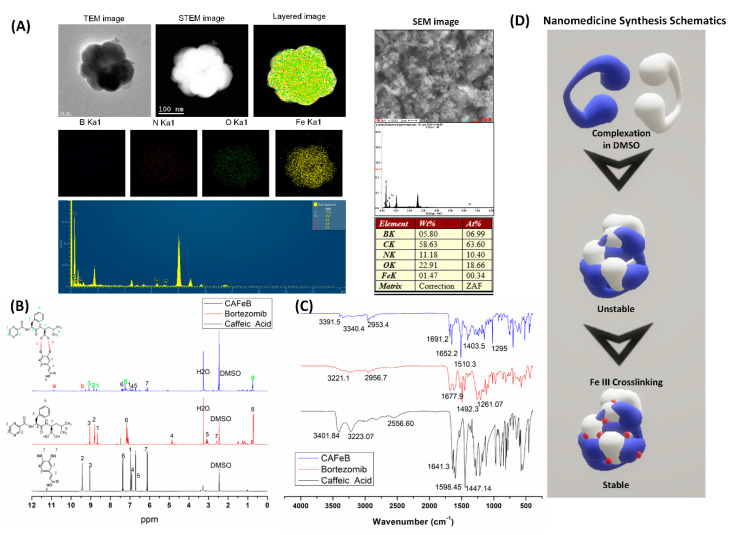
Characterization of the supramolecular prodrug nanomedicine. (**A**) Morphological assessment using FE-TEM, S-TEM, and SEM imaging and elemental analysis using EDS survey and mapping (boron, blue color; nitrogen, red color; oxygen, green color; iron, yellow color). (**B**) Proton NMR of caffeic acid, bortezomib, and CAFeB nanomedicine to validate the complexation process between the catechol and boronic acid moieties. (**C**) Functional group analysis of caffeic acid, bortezomib, and CAFeB. (**D**) Schematic of particle formation of CA and BTZ and the crosslinking process to stabilize the nanoparticle using Fe(III) ions.

The synthesized supramolecular prodrug nanomedicine had the following characteristics. The morphological structure of the prodrug nanomedicine was found to be an irregular spherical shape (Figure 2A). We verified the presence of the crosslinking ferric ions with the strong yellow signal from the elemental mapping. The FE-SEM/EDS data also verified the boron and Fe weight percentages at 5.8% and 1.47%, respectively, while the FE-TEM elemental spectrum showed around 8.4% and 0.6% for boron and ferric atoms, respectively. Much of the weight percentage of the prodrug nanomedicine was for carbon, oxygen, and nitrogen. These data initially verified the presence of BTZ and ferric ions in the synthesized nanomedicine.

Next, the presence of the functional groups and the conjugation of BTZ to CA needed to be confirmed, and this was achieved via H-NMR (Figure 2B) and FTIR (Figure 2C) spectroscopy analyses. The complexation of BTZ’s boronic acid functionality and caffeic acid’s catechol moiety could be observed with the presence of new ^1^H resonance at 10.9 ppm, compared with the BTZ and CA H-NMR spectra, where that resonance was absent (Figure 2B). The upshifting of some of the representative resonance in both CA and BTZ suggested that the protons had been de-shielded and there were more neighboring electronegative atoms, which in this case was oxygen. The FTIR spectra also corroborated the H-NMR data by confirming that the functional groups had been preserved, even after conjugation. The shifting of the peaks demonstrated hydrogen bonding within the molecule. However, since the synthesis method did not require any heating or highly polar solvents, we were able to make sure that the only change that had occurred was the complexation of BTZ to CA to make the supramolecular nanomedicine.

The stability of the supramolecular prodrug nanomedicine (CAFeB) was analyzed using DLS/zeta potential measurements, and we found that the size and zeta potential of CAFeB varied after submersion in PBS (pH 7.4) for 1, 2, and 4 weeks (Figure S1 of Supplementary Materials). This meant that the covalent bonds created between the macromolecules of BTZ and CA were hydrolyzed, but this happened very slowly, and when CAFeB was submerged in PBS (pH 4), the DLS measurements showed that the nanoparticle size had decreased abruptly and the zeta potential of CAFeB had changed from −28.7 ± −1.37 to −9.90 ± 1.1.3 eV (Figure S2 of Supplementary Materials, which was indicative of nanomedicine dissolution due to boronic acid and catechol complex dissociation.

Since the CAFeB nanomedicine can be considered as a prodrug with a drug and excipient formulation or a new drug entity in a nanoparticle formulation, it needed to be determined whether the components were all compatible with one another. We performed a thermal analysis using DSC-TGA to assess the heat flow and thermal degradation of the CAFeB nanomedicine, and compared it with pure BTZ and CA. Figure S3 of Supplementary Materials shows the DSC graphs for CA and BTZ, which revealed the characteristic exo/endothermic peaks corresponding to their melting point, an indication of the purity of the samples. However, in the CAFeB sample, we found that those peaks had disappeared, while the exothermic and endothermic peaks at 272.13 °C and 294.53 °C, respectively, had appeared. This was an indication of the crosslinking of the macromolecules, and the conjugation of BTZ to CA. With the higher binding energies of the components due to the dynamic covalent bonding of BTZ to CA and crosslinking of CA to Fe(III) ions, the heat flow could be seen to have shifted to higher temperature points. A shifting of peaks may indicate an incompatibility of the components, based on the conventional wisdom of drug and excipient formulations. Since the FTIR and H-NMR studies proved that the components still showed characteristic peaks of functional groups for CA and BTZ, it can be safely assumed that once the in vitro experiments are done, the biological effects of CA and BTZ can still be observed. The TGA analysis of CAFeB also indicated the thermal stability of the final nanomedicine with a final residue percentage of 56.17%. Pure CA and BTZ showed two thermal degradation points at 223.77–292.88 °C and 152.64–290.77 °C, respectively, while the CAFeB nanomedicine showed four thermal degradation peaks at 43.34, 164.56, 267.72, and 350.07 °C. The last two points were higher, compared with pure CA and BTZ. This was also in congruence with the dynamic covalent bonding between CA and BTZ and the crosslinking of CA-FeIII presented by other characterizations that were done in this study.

### 3.3. In Vitro Dose-Dependent Biocompatibility Analysis

To verify at which tolerable dose the nanomedicine is biocompatible, we conducted a dose-dependent analysis using 1–15 µg/mL of CA, BTZ, and CAFeB. Results showed that CA was biocompatible and enhanced the growth of NIH-3T3 fibroblast cells (Figure S4 of Supplementary Materials), which was due to the antioxidant effect of CA at lower doses [13]. On the other hand, BTZ had the most cytotoxic effect among the group, since it inhibits the 26S proteasome of the cells and could disrupt internal cell signaling in the recycling of damaged proteins. CAFeB also demonstrated the same effect, but with lesser average cytotoxicity at all doses studied, compared with free BTZ. This could be due to the nanoparticle formulation of CAFeB. Since it could take some time for the nanoparticle to be taken internally by the cells, the cytotoxic effects were delayed and less pronounced, compared with the pure molecular form of BTZ. These results were corroborated by the morphological analysis of fibroblast cells co-incubated with CA, BTZ, and CAFeB. The results showed that the cells incubated with BTZ and CAFeB had low cell counts, and the morphology was varied (Figure S5 of Supplementary Materials). The cells co-incubated with CA had normal morphology similar to the negative control, while BTZ and CAFeB had lesser cell proliferation and the morphology had deviated from normal as compared with the negative control. Nucleic staining using DAPI also verified the results of the CCK8 assay, and the nucleus of the cells incubated with BTZ and CAFeB showed increased blebbing, but CAFeB had lesser blebbing incidence compared with free BTZ. Another possible mechanism for the lesser cytotoxic activity of CAFeB is that normal fibroblast cells have lesser intrinsic ROS compared with malignant cells, and the CA component of CAFeB cannot create sufficient ROS damage in normal cells [27]. We expect to have increased cytotoxic effects in cancer cells because malignant cells are known to have higher intrinsic reactive oxygen species, due to their increased metabolic rate (Figure S6 of Supplementary Materials). CA can further increase this ROS level in cancer cells to create a higher degree of damage.

### 3.4. In Vitro CT26 Cancer Cell Line Antiproliferative Properties of the Nanomedicine

**Figure 3:**
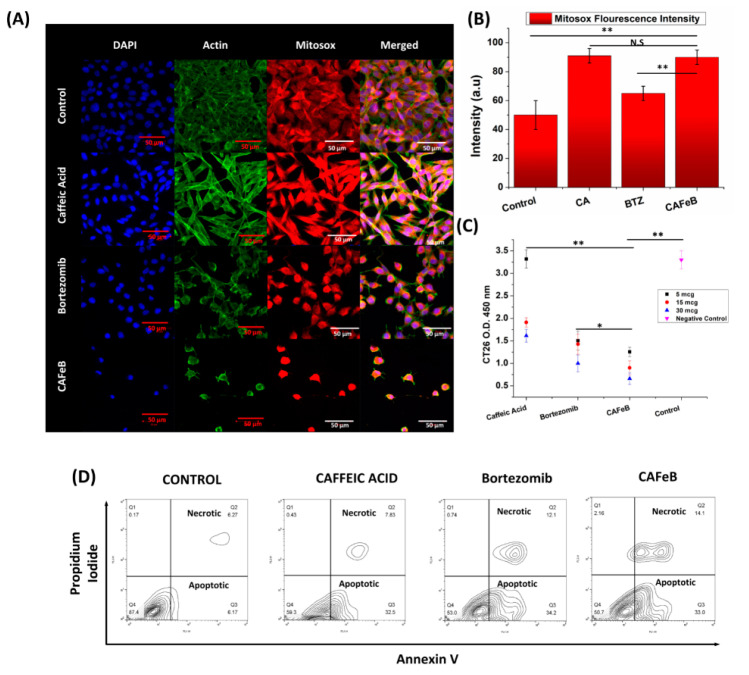
Analysis of the anticancer effects of caffeic acid, bortezomib, and CAFeB in vitro. (**A**) Confocal images of CT26 cancer cells using DAPI for nucleus staining, actin for cytoskeletal staining, and Mitosox Red for mitochondrial superoxide assessment under different co-incubation conditions (scale bar = 50 µm). (**B**) ImageJ fluorescence assessment of Mitosox fluorescence intensity. (**C**) CCK8 assay to determine the amount of metabolically active cells after 12 h under different co-incubation conditions (*n* = 3). (**D**) Representative FACS data determining the amount of apoptotic and necrotic CT26 cells using annexin V and propidium iodide staining.

To assess the anticancer properties of the nanomedicine in vitro, we chose CT26 mouse colon cancer as our model, due to its aggressive nature and fast-growing characteristics. We tested the metabolic activity of CT26 using the CCK8 assay (Figure 3C). The dosages of 30, 15, and 5 µg/mL of free CA, free BTZ, and the CAFeB nanomedicine were introduced to the CT26 cancer cells that were plated near confluence on a 96-well culture plate. After 12 h, the metabolic activity of the cells was measured, and the results showed that the cells co-incubated with CAFeB and free BTZ had an almost similar number of metabolically active cells, while free CA showed the least inhibiting property. In contrast to the main CCK8 assay, we performed a dose-dependent metabolic assay to assess how the CT26 cells will behave when exposed to the doses present in the nanomedicine at a 2.5:1 molar ratio. The results (Figure S11) showed that the cells exposed to CA had an absorbance lesser than BTZ, but greater than CAFeB. This indicated that CA at 21.4 mg/mL created a higher cytotoxic effect compared with BTZ at 8.56 mg/mL, and that using each component alone at doses similar to that present in the nanomedicine had less cytotoxic effects. However, we found that the antiproliferative effect of BTZ and CAFeB at low doses (µg/mL) were similar since the amount of CA at these dose levels cannot induce a high amount of ROS stress similar to higher doses of CAFeB (mg/mL). To corroborate the assay, we imaged the cells using DAPI and actin green for cell morphology assessment and Mitosox Red for mitochondrial superoxide determination (Figure 3A). The results showed that the cells co-incubated with free CA had normal morphological growth, while free BTZ and CAFeB exhibited cell distress via cytoskeleton stunting and nucleus blebbing. Mitochondrial ROS production was the highest in cells co-incubated with CAFeB, as shown in the confocal imaging and ImageJ analyses, indicative of the effect of CA pro-ROS effects (Figure 3B). The amount of apoptotic and necrotic cells was also determined via annexin V and propidium iodide staining (Figure 3D and Figure S9 of Supplementary Materials). CT26 cells that were co-incubated with free Ca, free BTZ, and CAFeB were analyzed using flow cytometry, which showed the distribution of CT26 cell fate of necrotic and apoptotic cells. The CAFeB group showed the highest population of necrotic and apoptotic cells (16.2 ± 2.2% and 34.2 ± 2.16%, respectively), followed by free BTZ (12.5 ± 2.2% and 30.0 ± 4.2%, respectively) and free CA (8.8 ± 2.2% and 32.2 ± 2.2%, respectively) (*n* = 3). These in vitro results verified our assessment that BTZ can be delivered inside the cells and it can exert anticancer effects by binding to the 26S proteasome of the cancer cells and inhibiting their survival mechanisms, while caffeic acid’s ROS-inducing effects damage intracellular proteins and DNA accumulation.

### 3.5. Cell Death Mechanism Study

**Figure 4:**
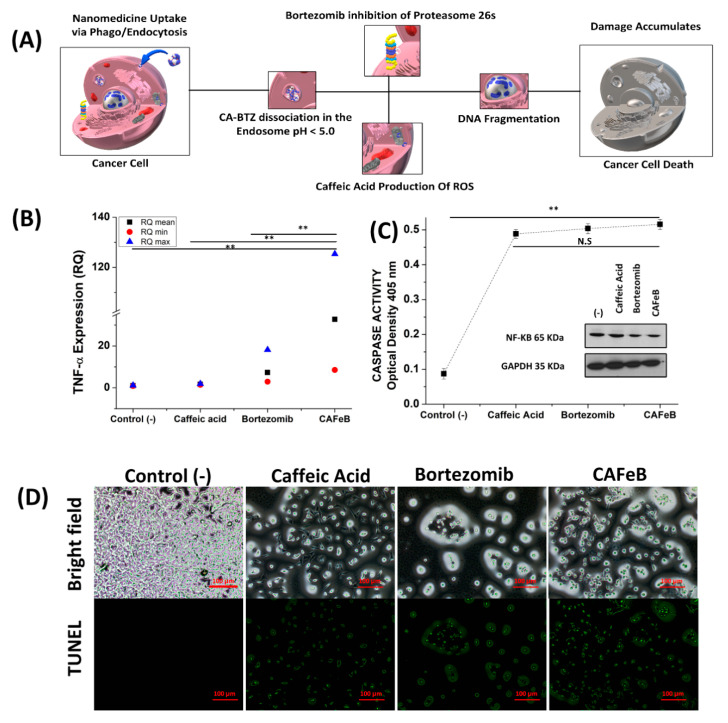
(**A**) Schematic of the cell death mechanism by the designed supramolecular nanomedicine. Confirmation of the cell death mechanism via (**B**) TNF-α expression quantitative PCR (*n* = 3), (**C**) caspase-3 activity assay (*n* = 3) (inset; western blot assay of NF-κB), and (**D**) DNA fragmentation visualization by TUNEL fluorescence staining (scale bar = 100 µm).

To verify the cell death mechanism hypothesis shown in Figure 4A, we conducted Q-PCR and western blotting to assess TNF-α cytokine levels and downregulation of NF-κB in CT26 cells after co-incubation with CA, BTZ, and CAFeB. As previously proven by Mitosox imaging (Figure 3A,B), the amount of mitochondrial ROS was elevated in cells that were exposed to caffeic acid. To enhance ROS damage inside cells, the ubiquitin–proteasome complex must be downregulated. As seen in Figure 4B, the TNF-α cytokine level was upregulated in the CAFeB group. TNF-α is involved in two complexes inside cells. The first complex leads to cell survival mechanism activation, and once it reaches a threshold, it would then activate the apoptosis signaling cascade. Increased production of TNF-α can also lead to activation of the cell survival mechanism of cancer cells, as it undergoes two sequential complexes. Complex I triggers rapid activation of transcription factors (nuclear factor-kappa B (NF-κB) and activator protein-1) and pro-survival genes, while complex II is formed after the former is released from the membrane into the cytosol by receptor internalization and endosomal trafficking. Complex II leads to death-inducing signaling complex formation, and ultimately, caspase-dependent apoptosis. NF-κB activation has been considered as the main pathway leading to differentiation and survival effects induced by TNF-α [28]; this was further corroborated by the western blot results, as CT26 cells co-incubated with BTZ and CAFeB had low levels of NF-κB, while cells co-incubated with CA had elevated NF-κB, due to the response of the cells to reactive oxygen species damage (Figure 4C, inset). Finally, DNA fragmentation was verified in CT26 cancer cells co-incubated with the CAFeB nanomedicine. The apoptosis response of the cells was observed through both the detection of nicked DNA through TUNEL staining and caspase-3 activity assay. The cell death mechanism can be observed based on morphological assessment, and biochemical and molecular changes of dying cells. The apoptotic activities through the caspase cascade were almost similar for bortezomib and CAFeB (Figure 4C), which meant that the CAFeB nanomedicine can disrupt the DNA of malignant cells, as caspase-3 is involved in internucleosomal cleavage or fragmentation [29]; TUNEL imaging and counting of TUNEL-positive cells confirmed this cleavage, as DNA strand breaks can be identified by labeling free 3′-OH termini with TUNEL fluorescein dyes (Figure 4D and Figure S10 of Supplementary Materials). These data confirmed that the nanomedicine’s cancer cell toxic properties by virtue of ROS damage and NF-κB inhibition resulted in apoptosis by DNA fragmentation and intracellular ROS damage.

## 4. Discussion

The designed nanomedicine had a unique combination of an ROS-inducing polyphenol (CA) and a 26S proteasome-inhibiting drug (BTZ). The synthesis of the nanomedicine followed a facile method that used a non-toxic solvent (DMSO), without the need for prolonged or complex chemical reactions. FTIR and H-NMR analysis proved the conjugation of CA and BTZ, and TEM elemental mapping analysis verified that Fe(III)-mediated crosslinking of the macromolecules was successfully performed. The natural affinity of the boronic acid functionality of BTZ to the catechol moiety of caffeic acid created a new supramolecular nanoparticle formulation, whose components complemented the effect of each other. The complexation was cleavable and reversible, depending on the pH of the system. With an acidic pH (< 5.0) the dynamic covalent bond between the catechol group of PCA and the boronic acid functionality of BTZ was protonated; thus, the BTZ payload was released from the nanoparticle, and the dissociation of the CAFeB nanomedicine was evidenced by the reduction of the particle size from 80 nm to 8 nm when exposed to acidic media (Figure S2 of Supplementary Materials). Furthermore, with the use of Fe(III) ions as a crosslinker to the complexed BTZ-CA macromolecules, the supramolecular structure of the nanoparticle could be stabilized. Without the Fe(III) crosslinker, the nanomedicine was unstable under a polar medium, which makes it impossible to use effectively inside the body. The main reason for making a stable nanoparticle from the supramolecular form of BTZ-PCA was that through the endocytic pathway, the acidic conditions in the endosome can be utilized and therefore, the BTZ can be liberated from CA, ensuring that the active site of the drug is free to inhibit the NF-κB-mediated cell survival mechanism of malignant cells. It has been recently discovered that BTZ is inhibited by naturally occurring polyphenols coming from natural diet. This nanoparticle formulation strategy shields the active boronic acid site of BTZ while in circulation and could increase its pharmacokinetic effects [26]. Another salient feature of the nanomedicine was the complementary effects of its components. The ROS-inducing properties of CA were demonstrated in this study and increased intracellular ROS can lead to apoptosis by damaging intracellular proteins. However, it is necessary to ensure that the ROS damages that the CA induces will not be checked by the cell’s ubiquitin–proteasome system, and that is where BTZ comes into action. As seen in the western blot studies in Figure 4, the downregulation of NF-κB was observed in both CT26 cells exposed to BTZ and CAFeB, but not with CA. The BTZ component of CAFeB selectively bound to proteasome 26S, inhibiting its ability to degrade unneeded or damaged proteins by proteolysis, a chemical reaction that breaks peptide bonds. Unchecked ROS inside the cell can now be had, creating a runaway cascade, and therefore having potent cancer-killing properties. However, due to the lack of a targeting moiety on the designed nanomedicine, we must ensure that it will pose lesser toxicity to the body when exposed to normal cells. We studied how NIH-3T3 fibroblast cells will react to the CAFeB nanomedicine and we found out that it created a slight cytotoxic effect, but due to the inherent lower levels of mitochondrial ROS on normal cells, oxidative stress did not reach apoptotic and necrotic threshold, thus exhibiting a lower cytotoxic effect compared with malignant cells (CT26) (Figures S4–S6 of Supplementary Materials). Lastly, this study further validated the combinatory effects of CA and BTZ reported by Altayli et al. [22], where comprehensive in vivo and in vitro studies were made using a multiple myeloma cell line. However, our nanoparticle formulation can improve the dosing and administration of both CA and BTZ by combining them into a single nanomedicine platform, potentially improving the pharmacokinetic behavior of both molecules once used in vivo.

## 5. Conclusions

A stable prodrug nanomedicine was developed using supramolecular chemistry. The conjugated caffeic acid and bortezomib can be cleaved when exposed to acidic environments, which are present in malignant microenvironments, and through endocytic and phagocytic intracellular uptake of nanoparticles, ensuring that both molecules can enact their roles. The nanomedicine was proven to have a combinatory anticancer property via caffeic acid’s reactive oxygen species induction and subsequent damaging properties in the malignant cells, while the bortezomib drug inhibited the cells’ ability to salvage damaged intracellular proteins created by the former. Finally, the supramolecular synthesis for this prodrug nanomedicine provides a new approach to creating a combinatory anticancer agent.

## Supplementary Materials

The following are available online at https://www.mdpi.com/1999-4923/12/11/1082/s1. Figure S1: Zetasizer analysis of the synthesized CAFeB prodrug nanomedicine (*n* = 3). The nanomedicine was dispersed in PBS (pH 7.4) and was subsequently analyzed under different time points. The size and zeta potential of CAFeB showed an inversely proportional relationship over time; Figure S2: pH-dependent analysis of the size and zeta potential of CAFeB using two different PBS conditions (pH 7.4 and 4.0). The results indicated that the size and zeta potential were dramatically reduced after the nanomedicine was dispersed in an acidic media; Figure S3: Thermal analysis (DSC-TGA) of pure caffeic acid, bortezomib, and CAFeB; Figure S4: Dose-dependent biocompatibility assessment of the CAFeB prodrug compared with pure caffeic acid and free bortezomib via CCK8 assay (*n* = 3); Figure S5: Biocompatibility assessment via morphological investigation and nucleus staining (DAPI); Figure S6: (A) Comparison of intrinsic mitochondrial ROS between normal fibroblast cells and malignant colon carcinoma and (B) fluorescence imaging using Mitosox Red fluorescence intensity analysis and ImageJ software; Figure S7: Caffeic acid dose-dependent biocompatibility assessment using (A) NIH-3T3 CCK8 assay graph (*n* = 3) and (B) bright-field imaging of the fibroblast cells; Figure S8: Densitometric analysis of western blot gels using ImageJ software and NF-κB expression in CT26 mouse colonic cancer cell line; Figure S9: FACS analysis of cell fate distribution using annexin and PI staining (*n* = 3 trials/10,000 events); Figure S10: TUNEL-positive cells (CT26) exposed to different treatment groups counted using ImageJ software (*n* = 3 image field); Figure S11: CCK8 metabolic assay of CT26 cells after dose-dependent exposure to CAFeB, caffeic acid (CA), and bortezomib (BTZ). Doses were calculated at a ratio of 2.5:1 mmol in relationship to the nanomedicine CAFeB.
