# Supplementary material for: Supramolecular Caffeic Acid and Bortezomib Nanomedicine: Prodrug Inducing Reactive Oxygen Species and Inhibiting Cancer Cell Survival
Source: Pharmaceutics. 2020 Nov 11;12(11):1082. doi: 10.3390/pharmaceutics12111082 (PMC7697389; doi:10.3390/pharmaceutics12111082)
Supplement: Supplementary file 1 [file pharmaceutics-12-01082-s001.pdf]

pharmaceutics

MDPI

Supplementary Information

Supramolecular Caffeic Acid and Bortezomib Nanomedicine: Reactive Oxygen Species Inducing and Cancer Cell Survival Inhibiting Prodrug.

Ludwig Erik Aguilar, Se Rim Jang, Chan Hee Park and Kang Min Lee

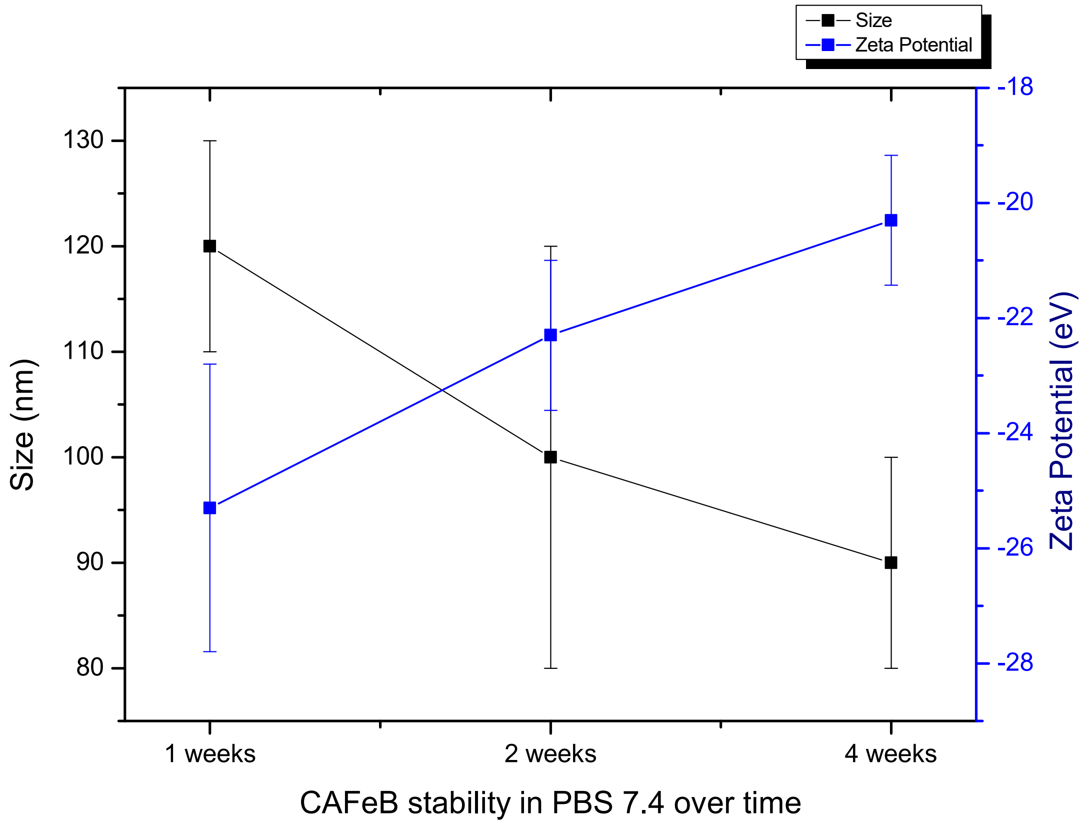

Figure S1. Zetasizer analysis of the synthesized CAFeB prodrug nanomedicine (n = 3). The nanomedicine was dispersed in PBS pH 7.4 and was subsequently analyzed under different time points. The Size and Zeta potential of CAFeB shows an inverse proportion over time.

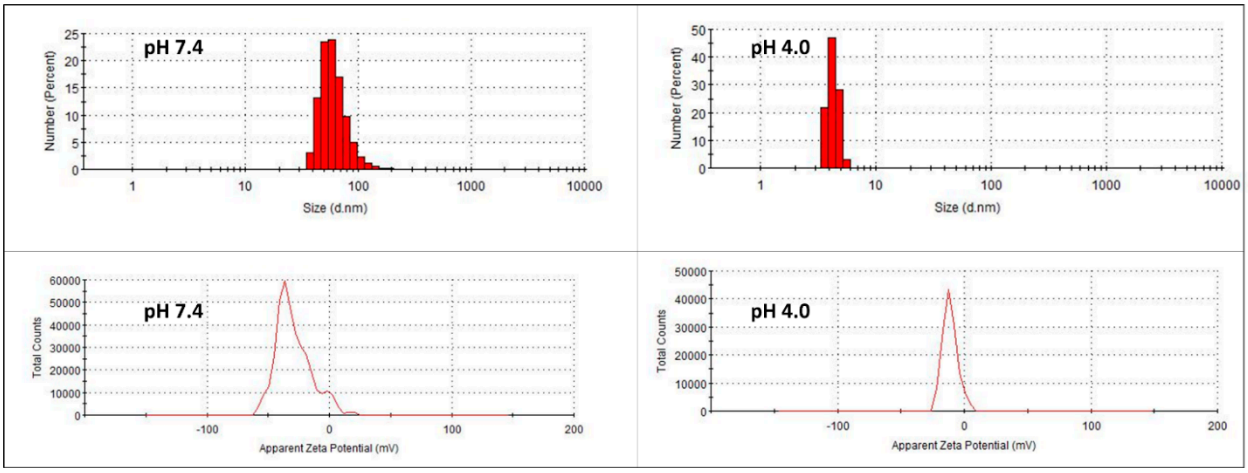

Figure S2. pH dependent analysis of the size and zeta potential of CAFeB using two diffent PBS conditions pH 7.4 and 4.0. The results indicate that the size and zeta potential has dramatically reduced after the nanomedicine was dispersed in an acidic media.

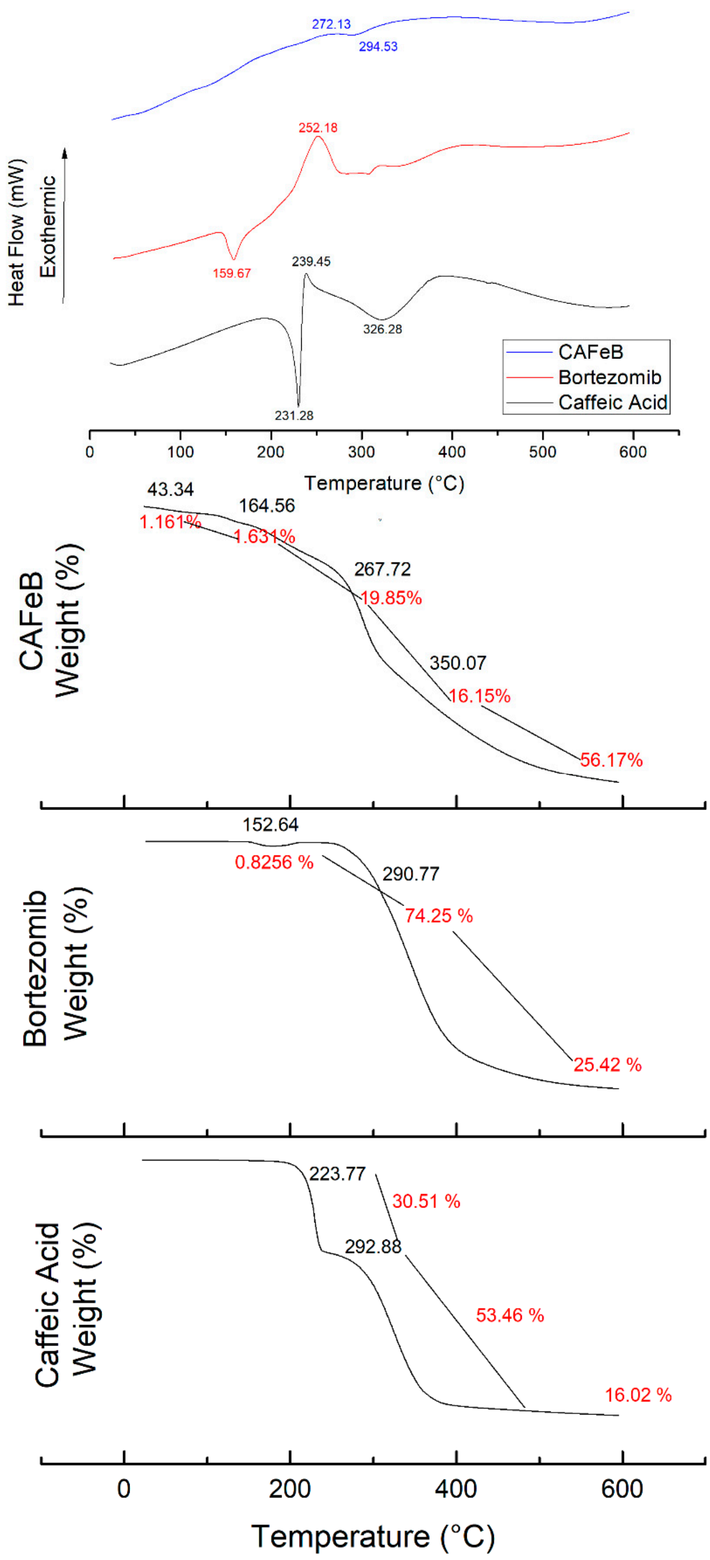

Figure S3. Thermal analysis (DSC-TGA) of the pure Caffeic Acid, Bortezomib and CAFeB.

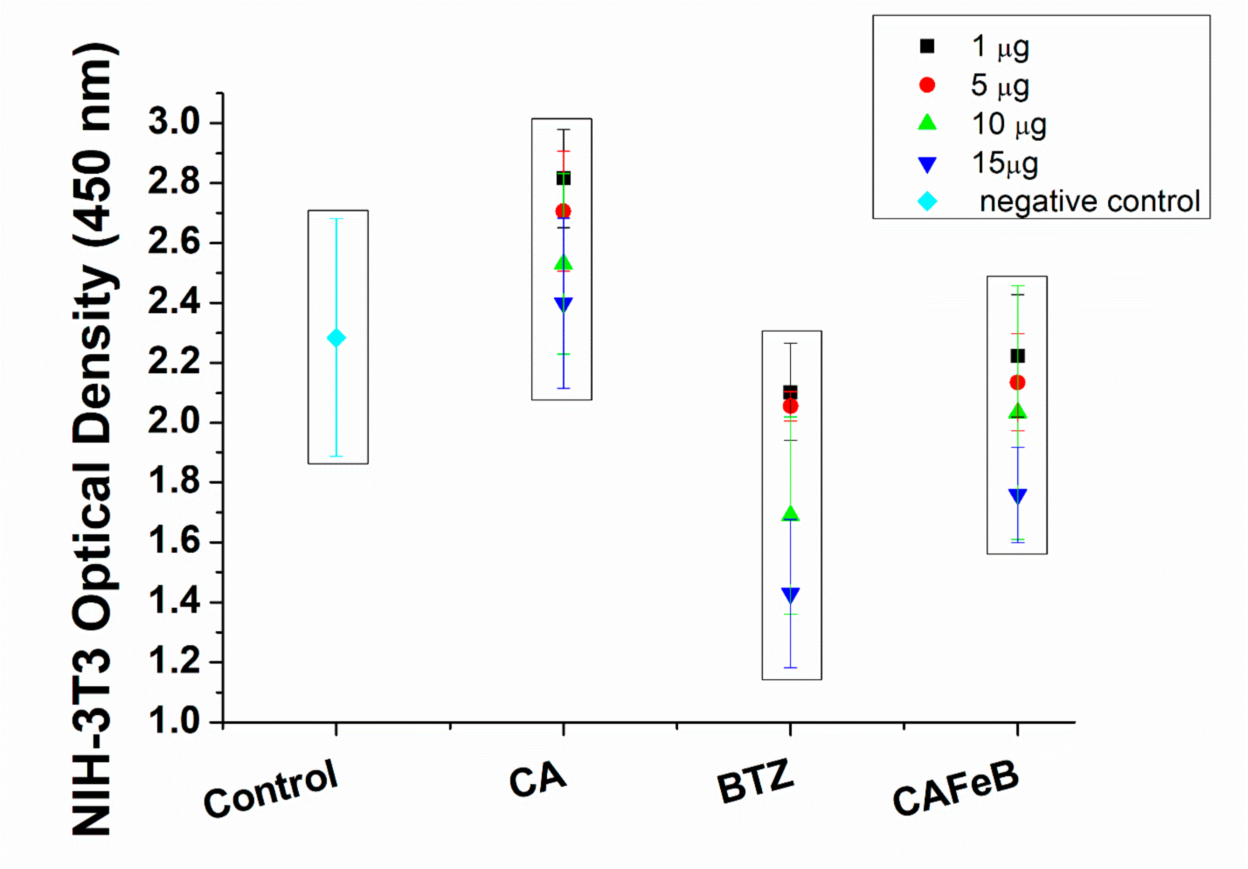

Figure S4. Dose dependent biocompatibility assessment of the CAFeB prodrug compared to pure Caffeic Acid and Free Bortezomib via CCK8 assay ($n = 3$).

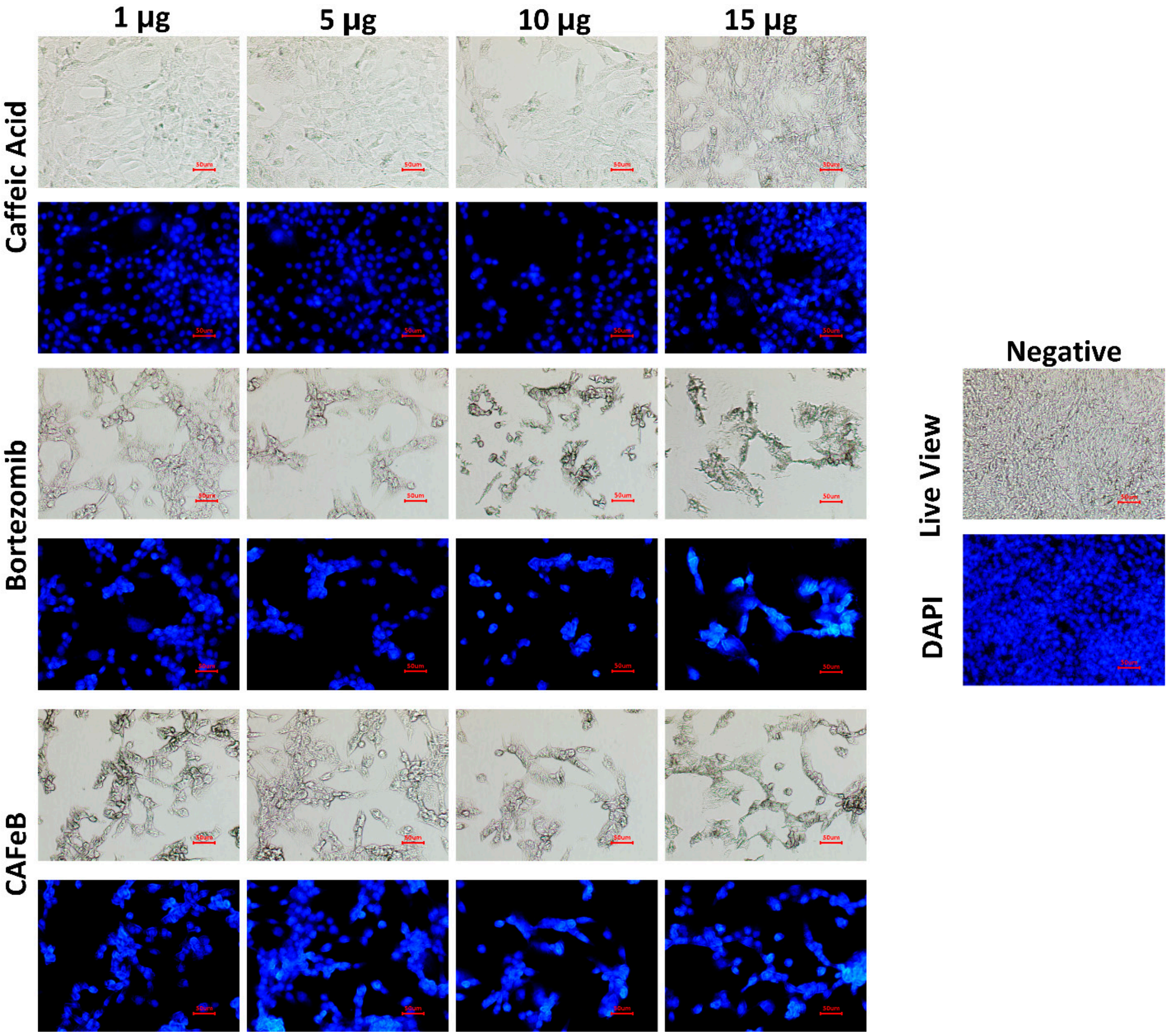

Figure S5. Biocompatibility assessment via morphological investigation and nucleus staining (DAPI).

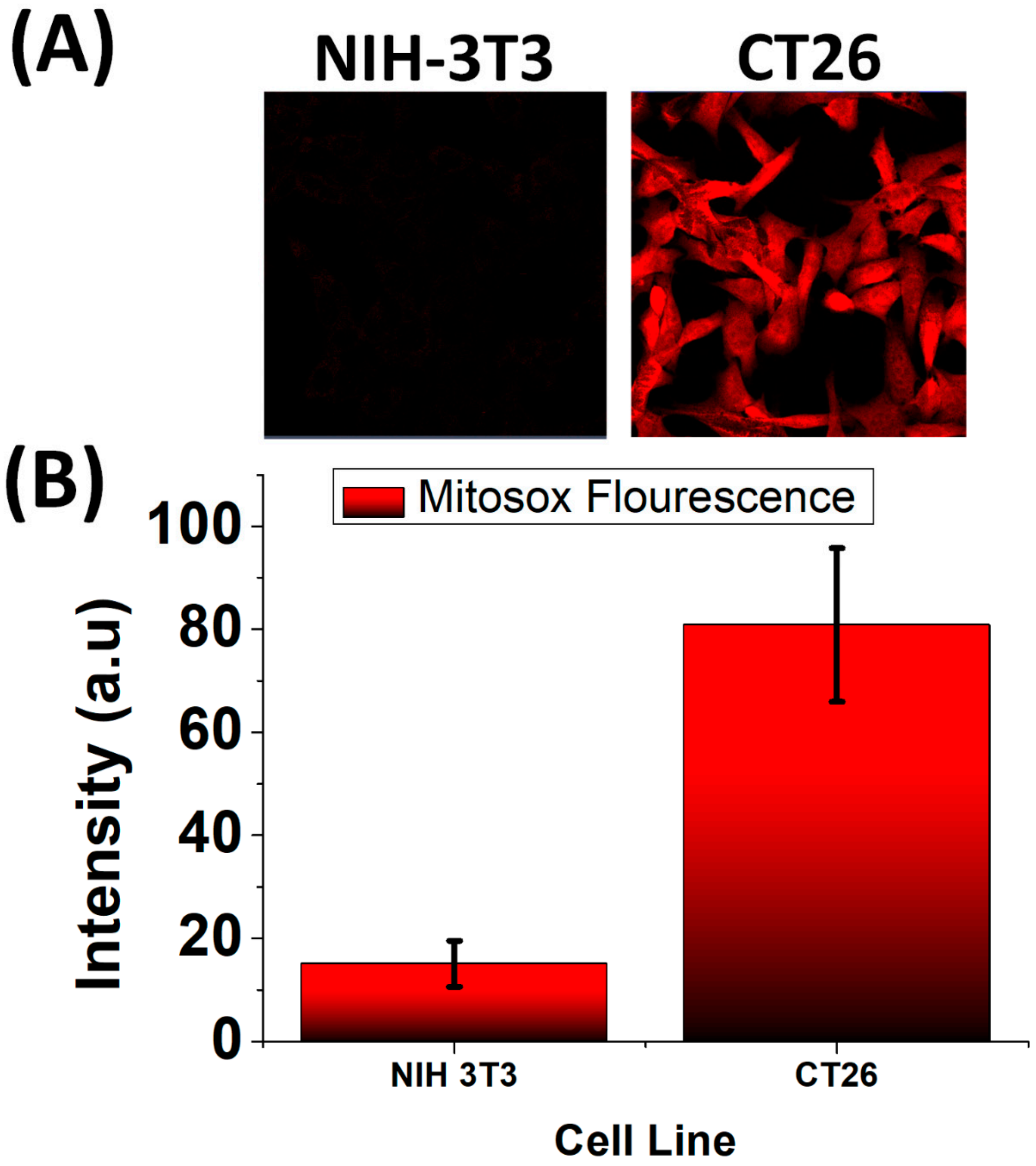

Figure S6. Comparison of intrinsic mitochondrial ROS between normal fibroblast cells and malignant colon carcinoma (**A**) Fluorescence imaging using Mitosox® Red (**B**) Fluorescence intensity analysis using ImageJ® software.

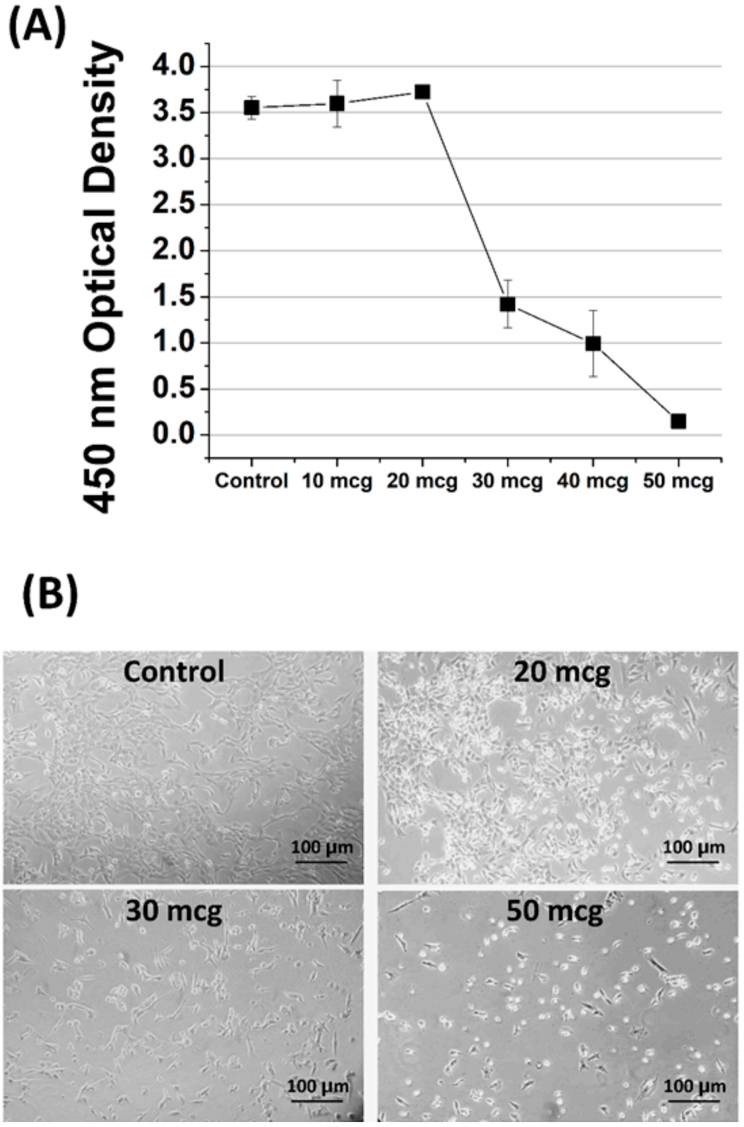

Figure S7. Caffeic acid dose dependent biocompatibility assessment using NIH-3T3 (**A**) CCK8 assay graph (n = 3) (**B**) Brightfield imaging of the fibroblast cells.

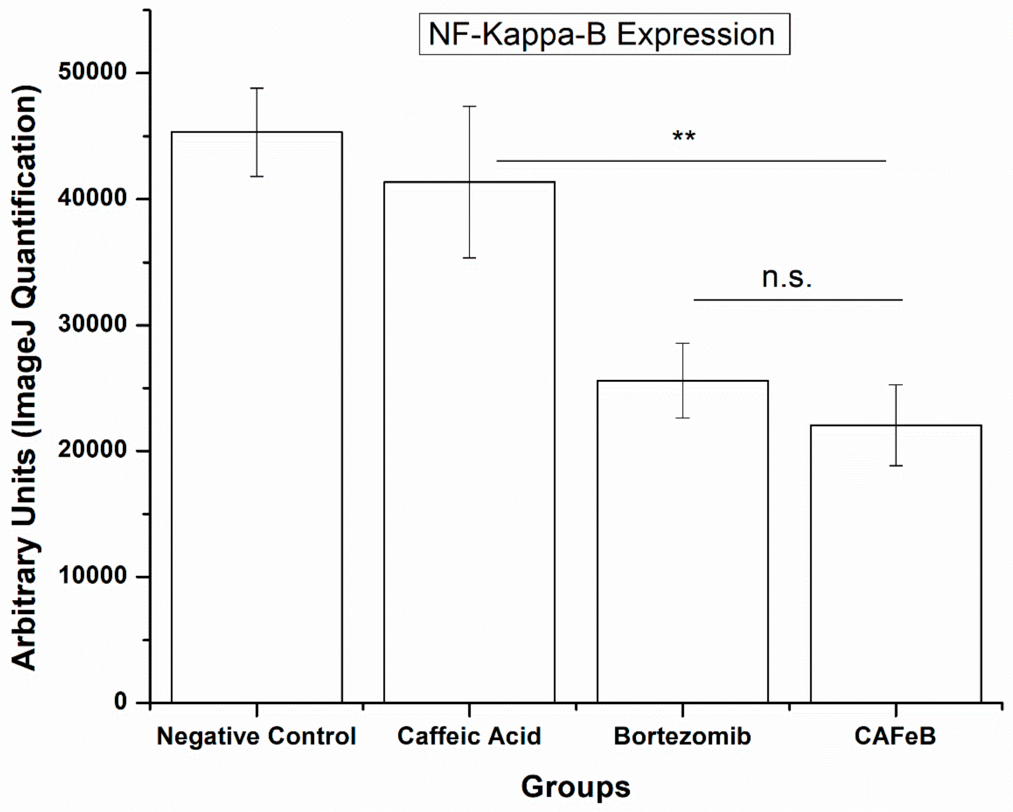

Figure S8. Densometric analysis of western blot gels using imageJ® software; NF-Kappa-B expression in CT26 mouse colonic cancer cell line.

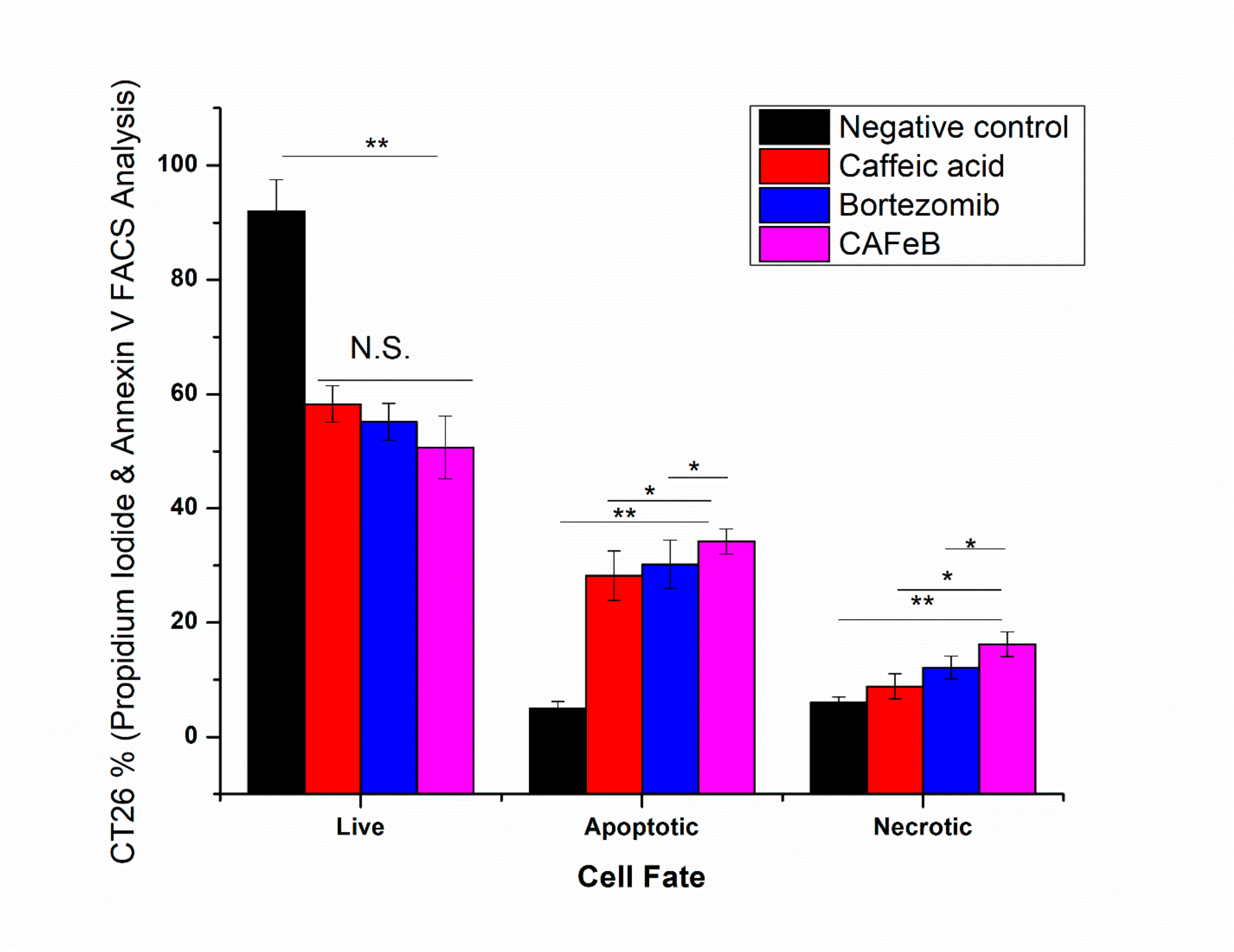

Figure S9. FACS analysis of cell fate distribution using Annexin & PI staining. (*n* = 3 trials/10,000 events)

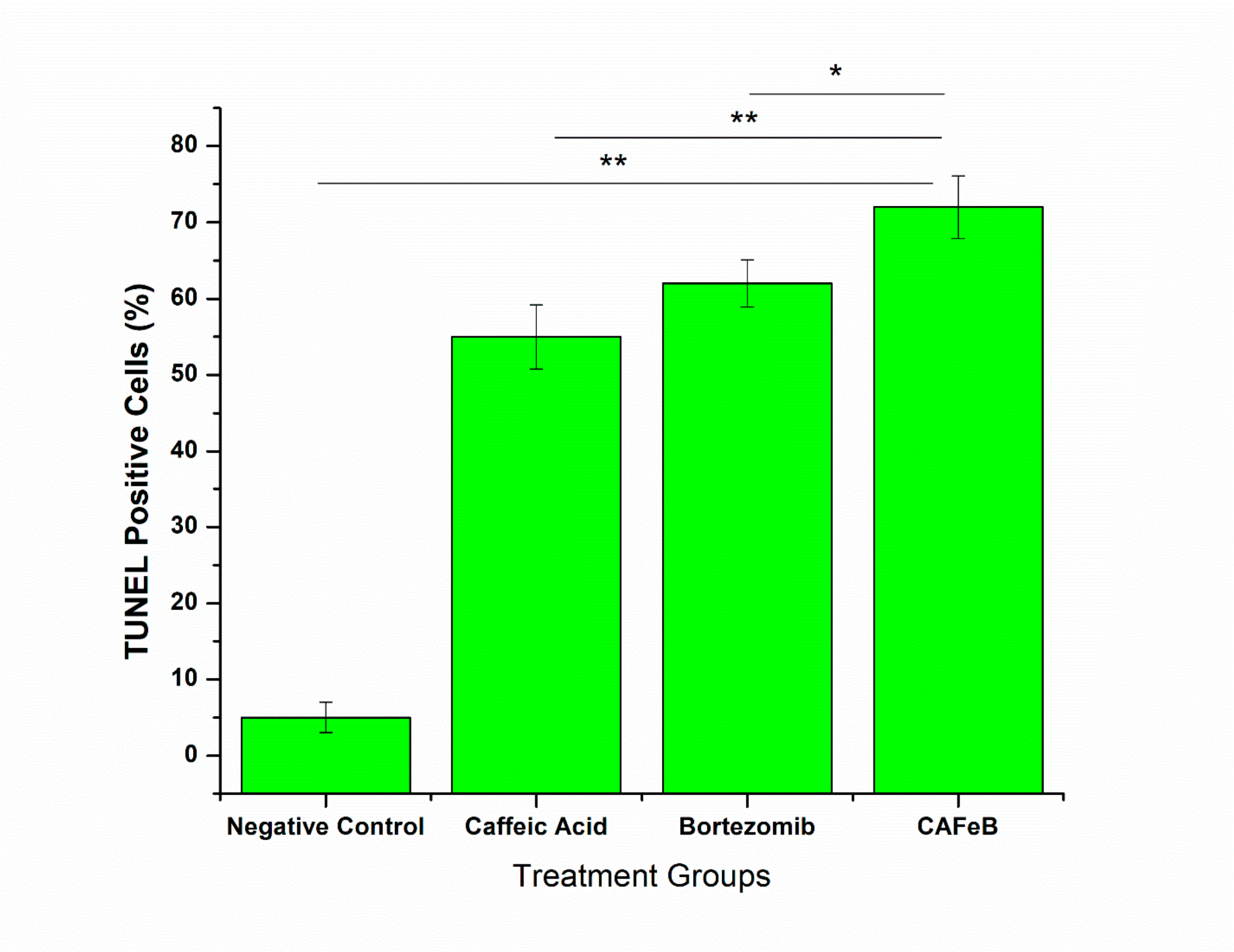

Figure S10. TUNEL positive cells (CT26) exposed to different treatment groups counted using imageJ® software (n = 3 image field).

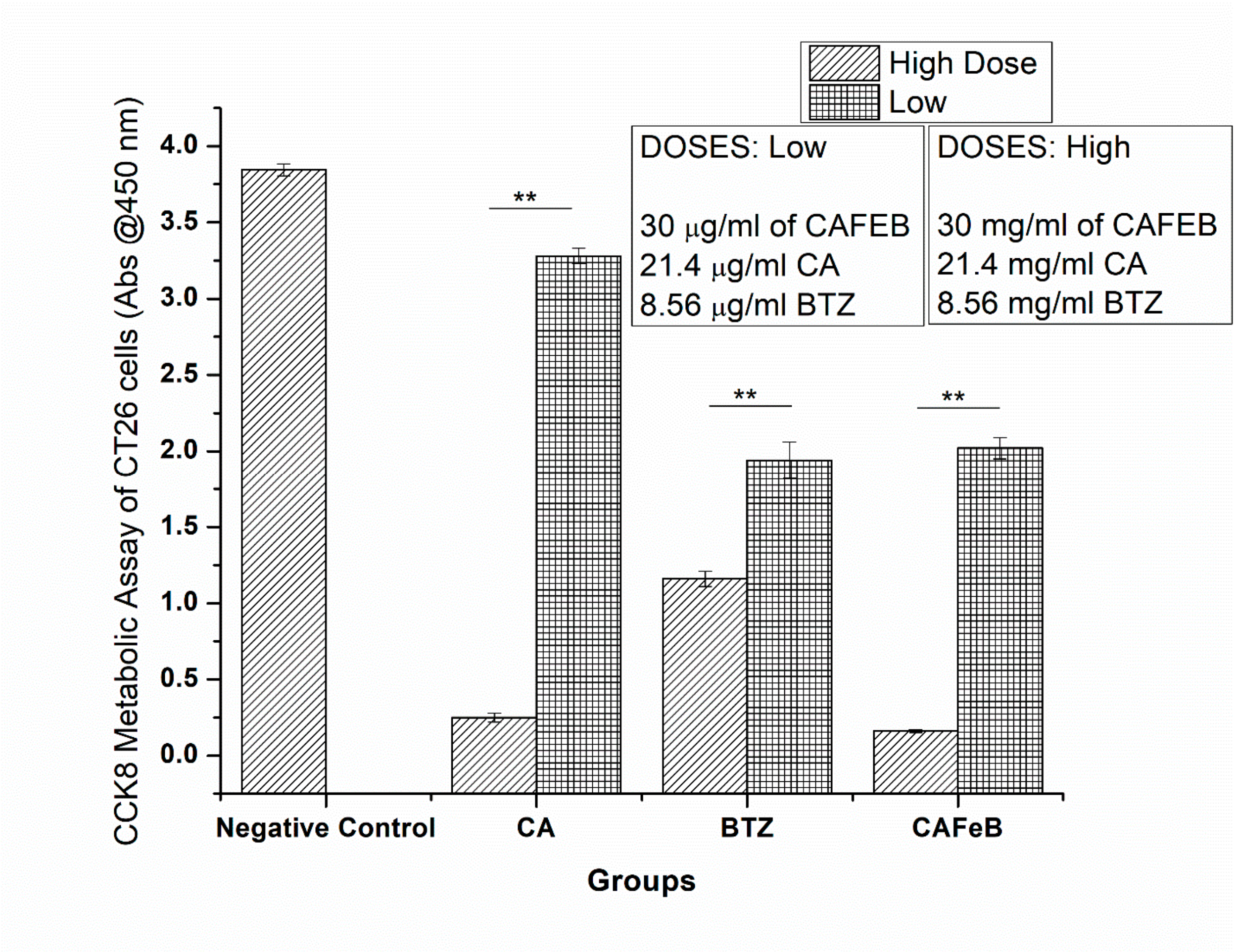

Figure S11. CCK8 metabolic assay of CT26 cells after dose dependent exposure to CAFeB, Caffeic Acid (CA), and Bortezomib (BTZ). Doses were calculated at a ratio of 2.5:1 mmol in relationship to the nanomedicine CAFeB.
